# The Quebec Low Back Pain Study: a protocol for an innovative 2-tier provincial cohort

**DOI:** 10.1097/PR9.0000000000000799

**Published:** 2019-12-19

**Authors:** Gabrielle M. Pagé, Anaïs Lacasse, the Quebec Back Pain Consortium (in alphabetical order), Nicolas Beaudet, Manon Choinière, Simon Deslauriers, Luda Diatchenko, Laurent Dupuis, Stéphanie Grégoire, Richard Hovey, Erwan Leclair, Guillaume Leonard, Carolina B. Meloto, Francesca Montagna, Alexandre Parent, Pierre Rainville, Jean-Sébastien Roy, Mathieu Roy, Mark A. Ware, Timothy H. Wideman, Laura S. Stone

**Affiliations:** a Department of Anesthesiology and Pain Medicine, Faculty of Medicine, Université de Montréal, Montreal, QC, Canada; b Centre de recherche du Centre hospitalier de l'Université de Montréal (CRCHUM), Montreal, QC, Canada; c Départment des sciences de la santé, Université du Québec en Abitibi-Témiscamingue, QC, Canada; d Quebec Pain Research Network, QC, Canada; e Department of Anesthesiology, Faculty of Medicine, Université de Sherbrooke, Sherbrooke, QC, Canada; f Centre de recherche du Centre hospitalier de l'Université de Sherbrooke (CRCHUS), Sherbrooke, QC, Canada; g Center for Interdisciplinary Research in Rehabilitation and Social Integration (CIRRIS, CIUSSS-CN), Quebec City, QC, Canada; h Department of Rehabilitation, Faculty of Medicine, Université Laval, Quebec City, QC, Canada; i Alan Edwards Centre for Research on Pain, McGill University Health Centre (MUHC), QC, Canada; j Faculty of Dentistry, McGill University, Montreal, QC, Canada; k School of Rehabilitation, Faculty of Medicine and Health Sciences, Research Center on Aging, Centre Intégré Universitaire de Santé et de Services Sociaux de l'Estrie—Centre Hospitalier Universitaire de Sherbrooke (CIUSSS de l'Estrie—CHUS), Université de Sherbrooke, Sherbrooke, Québec, Canada; l Centre de Recherche de l'Institut Universitaire de Gériatrie de Montréal, CIUSSS Centre-sud-de-l'île de Montréal, Montréal, QC, Canada; m Department of Stomatology, Université de Montréal, Montréal, QC, Canada; n Groupe de Recherche sur le Système Nerveux Central (GRSNC), and Centre de recherche en Neuropsychologie et Cognition (CERNEC), Université de Montréal, Montréal, QC, Canada; o Department of Psychology, Faculty of Science, McGill University, Montreal, QC, Canada; p School of Physical and Occupational Therapy, Faculty of Medicine, McGill University, Montreal, QC, Canada

**Keywords:** acute, chronic, persistence, inception cohort, low back pain, two-stage sampling

## Abstract

**Introduction:**

The neurobiological mechanisms underlying recovery from or persistence of low back pain (LBP) remain misunderstood, limiting progress toward effective management. We have developed an innovative two-tier design to study the transition from acute to chronic LBP. The objective of the first tier is to create a provincial web-based infrastructure to recruit and monitor the trajectory of individuals with acute LBP. The objective of the second tier is to fuel hypothesis-driven satellite data collection centers with specialized expertise to study the role of biomechanical, epigenetic, genetic, neuroanatomical, ontological, physiological, psychological, and socioeconomic factors in LBP chronicity.

**Methods:**

This article describes the first tier of the protocol: establishment of the Core Dataset and Cohort. Adults with acute LBP will be recruited through networks, media, and health care settings. A web-based interface will be used to collect self-reported variables at baseline and at 3, 6, 12, and 24 months. Acute LBP will be defined according to the Dionne 2008 consensus. Measurements will include the Canadian minimum data set for chronic LBP research, DN4 for neuropathic pain, comorbidities, EQ-5D-5L for quality of life, and linkage with provincial medico-administrative databases. The primary outcome will be the transition to chronic LBP, as defined by Deyo 2014. Secondary outcomes include health care resource utilization, disability, sick leave, mood, and quality of life.

**Perspective:**

This study brings together diverse research expertise to investigate the transition from acute to chronic LBP, characterize the progression to recovery or chronicity, and identify patterns associated with that progression.

## Abstract 2

Supplemental Digital Content is Available in the Text.

## 1. Introduction

The estimated worldwide 1-month prevalence of low back pain (LBP) is as high as 23%, suggesting that about a quarter of the global population experiences LBP in any given month.^25,32^ According to a recent systematic review, the lifetime prevalence of LBP ranges from 2% in urban adults aged ≥15 years in Pakistan to 86% among adults (≥18 years) in Germany.^25^ Low back pain is also the leading cause of global years-lived-with-disability.^44^ The high prevalence of LBP has significant economic consequences, with direct medical cost estimates ranging from 12.2 to 90 billion US dollars annually.^10^ Low back pain is the sixth most costly disease, behind ischemic heart disease, motor vehicle accidents, acute respiratory infections, arthropathies, and hypertension.^13^

Most LBP cases cannot be linked conclusively to a specific nociceptive source or pathoanatomical cause,^31^ and factors associated with LBP chronicity remain poorly understood.^2,21,27^ Although some risk factors have been extensively studied (individual/socioeconomic, occupational, pain characteristics, and psychological),^5,18,30,33,34^ others have not (biomechanical, epigenetic, genetic, and neuroanatomical). A better understanding of the vulnerabilities underlying recovery or persistence of LBP will allow for early intervention and prevention of the progression to a chronic state.

Many regions or countries are developing acute LBP cohorts including Australia, United States, and Denmark.^9,22,28,35^ Although comprehensive longitudinal data can be derived from these cohorts, limitations exist. For example, recruitment of participants from a limited number of clinicians located in a restricted geographical region excludes individuals who have not seen a professional, who have seen a professional not participating in the cohort study, or who live outside the designated region. A number of additional large^8,16^ and smaller^14,15^ prospective cohorts have initiated recruitment without a clear focus on any particular stage of LBP. Finally, other large cohorts have been built to identify the occupational as well as the sociodemographic, general health, and psychological risk factors for the occurrence of chronic LBP without a clear strategy for the integration of these factors with biomechanical, epigenetic, genetic, and neuroanatomical characteristics.^19,26,39^ A unifying project, focused on the transition from acute to chronic LBP that brings together basic, clinical, and epidemiological methods/expertise, is needed to understand the complex nature of these factors and their interactions.

The Quebec Low Back Pain Study (QLBPS), a strategic initiative of the Quebec Back Pain Consortium (QBPC; https://backpainconsortium.ca) and the Quebec Pain Research Network (QPRN; https://qprn.ca/en), was created to address these limitations. The proposed design enables the integration of information across multiple domains, thus providing a comprehensive view of risk factors for LBP chronicity. The scientific committee of the QBPC is composed of experts from diverse disciplines including physical therapy and biomechanics, epigenetics, genetics, neuroanatomy, medicine, epidemiology, and health ontology and psychology, in addition to patients' perspective representatives. The long-term goal of our consortium is to establish a province-wide online database for longitudinal studies of individuals with LBP. This will provide a comprehensive view of the risk factors for the development of chronic LBP that will break disciplinary silos.

## 2. Methods

This multidisciplinary, longitudinal cohort protocol has been prepared according to the SPIRIT 2013 guidelines for completeness and quality of trial protocols^7^ and complies with the Strengthening the Reporting of Observational Studies in Epidemiology (STROBE) statement.^42,43^

### 2.1. Context

**Figure 1.**
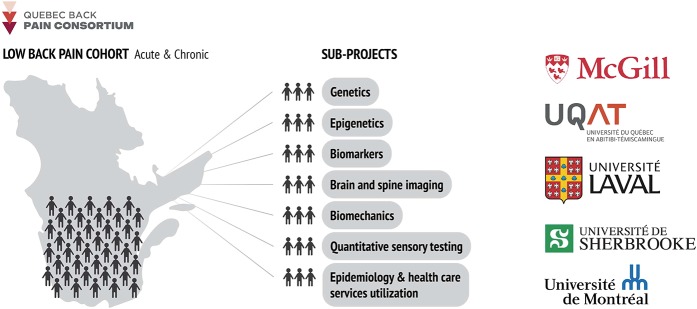
Quebec Back Pain Consortium vision and participating members.

The QLBPS was developed by the QBPC, a strategic initiative of the QPRN funded by the *Fonds de Recherche du Québec—Santé*, a provincial government funding agency. The scientific committee has been meeting regularly since 2015 to develop a shared vision and to design and implement the study described here. Figure 1 illustrates the vision and participating institutions.

### 2.2. Study design

**Figure 2.**
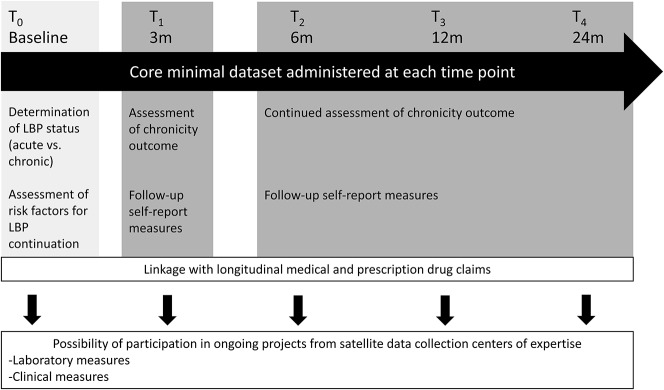
The Quebec Low Back Pain Study methodology. T0 to T4 represent the different data collection time point for all patients enrolled. Everyone will complete the core minimal data set 5 times within the period of 2 years. The baseline questionnaires will be used to categorize patients into acute or chronic LBP and identify risk factors that predict the maintenance of the LBP. At 3 months and for all subsequent time points, LBP status will be re-evaluated along with secondary outcomes. When patients consent, the provincial health plan number (RAMQ, Régie de l'assurance maladie du Québec) will be collected to merge with their medical and prescription drug claims. Finally, at any point during their active participation in the study, patients could be solicited to participate in one or more of the ongoing satellite projects. LBP, low back pain.

We will use a 2-stage approach (Fig. 2). The first stage, described here, will create the QLBPS Core Dataset and Cohort. Self-reported variables will be assessed in this provincial sample of patients suffering from LBP with follow-ups at 3, 6, 12, and 24 months after baseline. The Cohort will then “fuel” smaller studies in the second stage where patients will be invited to visit specific research laboratories for more extensive measurements (eg, biomechanics, epigenetics, genetics, neuroimaging, and sensory testing). All data collected from the first stage will be available for the satellite projects conducted in the second stage.

### 2.3. Inclusion and exclusion criteria

Potential participants will be 18 years and older, have internet access, be fluent in either French or English, and have self-reported LBP. No specific exclusion criteria will be applied.

### 2.4. Recruitment

Recruitment was initiated in November 2018 and is ongoing; 46 acute LBP patients completed the baseline questionnaires in the 7-month period between November 2018 and June 2019. In consultation with marketing experts, we then optimized of our recruitment strategy, resulting in 7 additional acute LBP patients weekly between June 2019 and August 2019. The recruitment strategy includes the following target populations: (1) general population in the province of Quebec (through advertisement in newspapers, public means of transportation, and social media such as Facebook); (2) individuals or institutions by email list servers; (3) populations of individuals at risk of LBP (through advertisement with patient advocacy organizations or professional societies [ie, *Association québécoise de la douleur chronique*, QPRN, Canadian Pain Society]); (4) unions of workers at risk for LBP (including but not limited to construction workers, warehouse workers, public service workers [ie, police and firefighters], nurses, other allied health professionals, and bus drivers); and (5) medical populations (through advertisements in emergency units, medical, physiotherapy, chiropractic, radiology clinics, and pharmacies).

In parallel to the acute-phase recruitment, individuals with chronic LBP who self-register for the study will be included in the Core Database. Their longitudinal data will be valuable for comparative purposes, for additional trajectory analyses, and for recruitment of chronic LBP participants in affiliated satellite projects.

### 2.5. Data collection methods

The recruitment strategies listed above will all include an invitation to a web platform (https://backpainconsortium.ca/), and/or aliases mybackhurts.ca or malaudos.ca. On this platform, interested participants will be asked to provide their postal code, sex, age, pain location, and intensity. Potential participants will confirm their interest by providing their email and full name and will complete a CAPTCHA (Completely Automated Public Turing Test To Tell Computers and Humans Apart) verification. Only those providing all the required information and that self-identify with LBP will receive an email inviting them to access the baseline online survey. This email will also include a link to the informed consent form, will encourage potential participants to read the document carefully, and will inform them that the study coordinator is available for any questions. Participants will be enrolled after they electronically provide their consent to participate. They will also be asked whether they are willing to be contacted again by other researchers and/or if they are willing to donate a blood sample. Once enrolled, they will be automatically redirected to the web-based baseline self-administered questionnaire. Baseline and follow-up questionnaires are hosted on the data collection platform Research Electronic Data Capture (REDCap) for electronic collection and management of research data. Completion of the baseline questionnaire takes approximately 20 minutes to complete. At 3, 6, 12, and 24 months after completing the baseline questionnaire, participants will receive an email with a unique link for completion of follow-up web-based questionnaires that take approximately 20 minutes to complete.

Evidenced-based retention strategies^1,20,36^ will be applied. At each time point, participants who do not complete the above-mentioned questionnaires will be contacted by the study coordinator by phone and/or e-mail no more than 3 times. Other retention strategies include sending a quarterly newsletter to recruited participants to inform them of study progress, to share relevant information and resources about LBP, and to “humanize” the research by bridging the gap between researchers' goals and participants using photographs and biographies, for example. Participants will have the potential to share their experience with other participants through the newsletter and will receive tokens of appreciation as they complete steps in the study such as a pen, a fridge magnet, or a badge. Finally, all participants will be invited to enter into drawings for prepaid VISA cards.

### 2.6. Study variables and validated measurements

Multiple meetings between the authors and a thorough review of the existing literature on LBP and psychometric measurements were conducted to select the core data set. Criteria for inclusion in the core data set was based on recommendations from expert research groups, including the National Institutes for Health Research (NIH) Task Force on Research Standards for Chronic Low Back Pain^11^ and the IMMPACT consensus recommendations.^41^ Measures included in the core data set are presented in Table 1. A trick question (please select number 4 in the options below) has been inserted to gauge the quality of participants' responses. Permission has been obtained to use the NIH, DN4, and EQ-5D-5L questionnaires for research purposes. The complete set of questions is included in Supplementary Appendix 1 (available as supplemental digital content at http://links.lww.com/PR9/A59). The psychometric properties of the scales are included in Supplementary Appendix 2 (available as supplemental digital content at http://links.lww.com/PR9/A59).

**Table 1:**
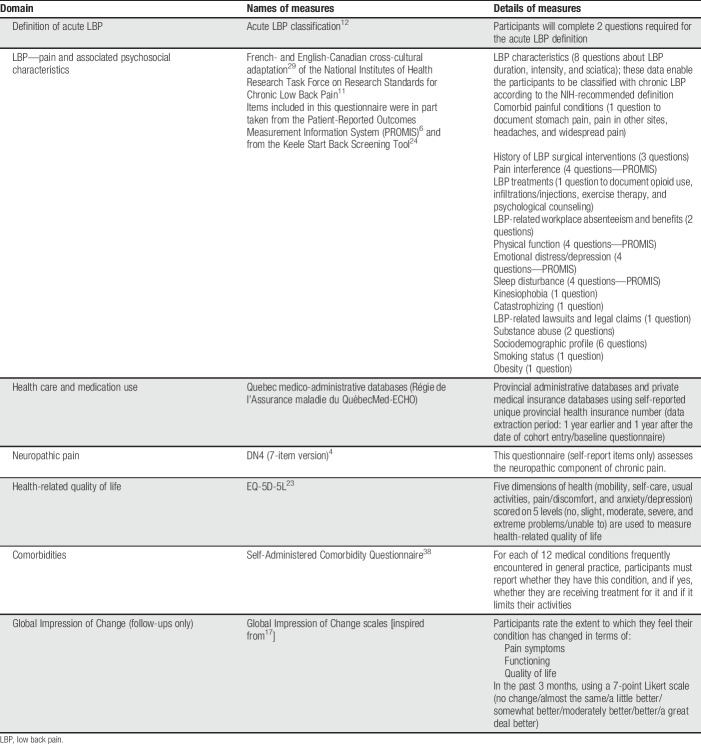
Details of measures and variables included in the minimal core data set.

### 2.7. Characterization of low back pain status

The criteria for acute LBP will be based on the consensus statement of Dionne and colleagues.^12^ According to their recommendations, patients who answer “yes” to the 2 following questions are suffering from acute LBP: (1) In the past 4 weeks, have you had pain in your lower back (in the area shown on the diagram), and (2) if yes, was this pain bad enough to limit your usual activities or change your daily routine for more than one day?

### 2.8. Primary outcome

The primary outcome of the Core Dataset is whether participants who reported acute LBP at baseline will transition to chronic LBP. Chronic LBP will be defined based on the NIH task force recommendations that define chronic LBP as an ongoing problem for at least 3 months and that has resulted in a problem on at least half of the days in the past 6 months.^11^

### 2.9. Secondary outcomes

Secondary outcomes include the duration of the LBP episode and/or the presence (and number) of recurrent LBP episodes,^40^ work status, use of health care resources and direct medical costs incurred (including economic burden data), participants' functional limitations and sick leave, mood, as well as the longitudinal trajectory of health-related quality of life.

### 2.10. Data analysis (Core Dataset)

All study variables will be screened for normality as well as univariate and multivariate outliers. Missing data will be handled with multiple imputation when indicated.^37^ Mean values, medians, and SDs will be computed for continuous variables while percentages and frequencies will be computed for categorical variables. Depending on the distribution of variables, longitudinal changes in pain severity and their predictors will be examined using various multivariate approaches, including latent class analysis or hierarchical cluster informed by linear regression. A *P*-value ≤0.05 will be considered statistically significant.

The QLBPS Core Cohort is a continuous recruitment platform; thus, no minimum or maximum number of participants has been set.

### 2.11. Ethical approval and confidentiality

The QLBPS Core Dataset (first tier) has received ethical approval from McGill University (A06-M22-18A) and will be conducted in conformity with the ethical principles set forth by the Regulatory Framework in Health Research at the McGill University Health Centre in accordance with the second edition of the Tri-council Policy Statement. Participants will be automatically assigned a study ID by REDCap ensuring that only the principal investigator and study coordinators will have access to participants' ID. Nominal data and study ID numbers are kept in a separate Excel file protected by a password. All other study investigators and staff will have access to denominalized data. Any important protocol modifications will be subject to approval from the scientific committee of the Quebec Back Pain Consortium and the Ethics Committee of McGill University.

### 2.12. Second-stage sampling

In the baseline online survey, participants will be asked whether they are willing to be contacted for participation in additional studies, where they live and whether they would consider donating blood. The answers will be recorded in the Core Database and will be used to identify participants who may be contacted by other QPRN-approved investigators for satellite data collection centers. If participants are interested in donating blood, they will receive an email from the QLBPS Biobank Team. The biobank is covered under a separate sister protocol at the McGill Ethics Committee entitled “Quebec Low Back Pain Biobank” (A08-M23-18A).

### 2.13. Satellite projects

Researchers with ethical approval at their home institutions and from the scientific committee of the QLBPS will be provided participants' study ID, name, and contact information. This is the only time that investigators will have access to nominalized data and will be for recruitment purposes only. All other internal and external researchers will have access to the deidentified QLBPS Core Dataset upon request. A current list of scientific committee-approved satellite projects is shown in Table 2.

**Table 2:**
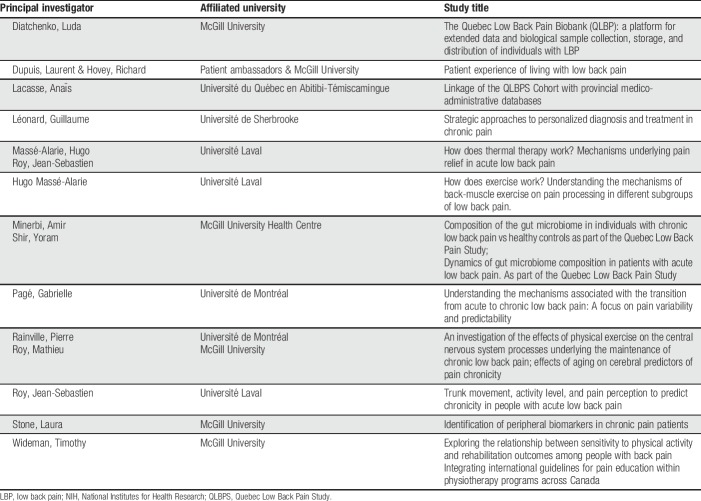
Satellite projects affiliated with the Quebec Low Back Pain Study.

### 2.14. Assignation and allocation rules for the satellite projects

Participants who agreed to be contacted to participate in additional studies will be assigned to ongoing satellite projects associated with the QLBPS based on each project's inclusion and exclusion criteria. As part of the consortium, researchers of ongoing satellite projects will have a shared script and recruitment efforts will be coordinated by the consortium study coordinator. This shared workflow will optimize the utility of the cohort and minimize burden to patients. Potential bias to the dispatch process of participants into satellite projects include geographic location of participants, which may limit the projects they can enroll in, and participants' willingness to undergo procedures such as blood donation or magnetic resonance imaging. Bias in the assignment to satellite studies that are in competition for subjects will be mitigated by the shared scripts and managed by the coordinator. More specifically, participants meeting the criteria for more than one satellite project will be assigned to projects in turn; priority will alternate between each project, taking into account the targeted sample size.

### 2.15. Dissemination

Results from the Core Dataset as well as those from satellite projects will be presented at national and international conferences and published in peer-reviewed journals in the fields of pain and musculoskeletal diseases, as well as discipline-specific journals relevant to the specific questions and methodologies of the satellite projects.

## 3. Discussion

A multidisciplinary approach investigating the factors contributing to the recovery from LBP, the recurrence of LBP episodes, and the factors influencing the transition to chronic LBP will be facilitated by the pragmatic two-stage sampling approach where patients with acute LBP are continuously enrolled in a core longitudinal cohort and subsequently recruited for satellite projects tackling specific questions. Given the high global incidence and costs of LBP,^10,25,32^ improved understanding of the mechanisms driving LBP chronicity is desperately needed.^21,27^

This study design confers multiple advantages. First, the existence of the Core Dataset and the composition of its scientific committee will allow for the examination of the chronicity of LBP from a large array of research perspectives including biomechanical, epigenetic, genetic, neuroanatomical, ontological, psychological, physiological, and socioeconomical mechanisms. This approach will make it possible to examine simultaneously the multifactorial determinants of LBP by providing adequate statistical power and measurement. Second, a common set of carefully selected measures assessed from the acute phase (baseline) up to 2-year follow-up will be available to all researchers. These measures are based on the minimum data set developed by the NIH Task Force on Research Standards for Chronic Low Back Pain^11^ and on our Canadian adaptation of those recommendations.^29^ Third, the Core Dataset will include participants from the entire province of Quebec, ensuring representation from urban and rural areas and remote regions. Given its web-based format, the design also allows for expansion nationally and internationally. Fourth, for participants who provide their unique provincial health insurance number, health care use, including medication, can be studied to assess important aspects of the economic burden of LBP. Finally, the design provides a practical solution for deep phenotyping of a subset of participants while maintaining the sample sizes needed for epidemiological studies.

Some limitations must nonetheless be noted. First, no formal medical validation of participants' LBP or its potential drivers will be included for the Core Dataset as all measures will be self-reported. However, medical validation will be possible in satellite projects. Second, there will be geographical barriers to participation in satellite projects such as the physical distance from research laboratories with specialized equipment or expertise (eg, MRI facilities specialized in brain and spinal imaging). Third, to keep the questionnaire short, not all domains are fully explored. For example, although the NIH section includes several questions on employment and legal status, job satisfaction is not assessed. Finally, the Core Dataset is available online through a web-based platform; participation may therefore be difficult for individuals with limited computer skills. This concern is mitigated by (1) availability of our staff to guide participants by phone and (2) data showing that 88% of individuals in Canada have access to Internet for personal use.^3^

Ultimately, the Core Dataset will provide: (1) a pool of potential LBP patients for other research studies, (2) a database allowing for investigation s into acute to chronic LBP, and (3) analysis of the relationships between parameters collected as part of the Core and affiliated studies, such as pain trajectories and blood-based genetic or epigenetic factors.

In summary, the QLBPS Core Cohort and Dataset, with its innovative two-stage sampling approach, will constitute a valuable platform for the continuous enrolment of LBP patients to facilitate the integrated investigation of factors (eg, biomechanical, epigenetic, genetic, neuroanatomical, ontological, physiological, psychological, and socioeconomic) contributing to the transition from acute to chronic LBP, how these factors might change with time as pain progresses, and what are the health care utilization and medication patterns associated with that progression. The integration of data gathered from the QLBPS Core Dataset, and the satellite data collection centers of expertise will support the development of comprehensive profiles of patients at risk of LBP chronicity.

## Disclosures

L. Diatchenko and F. Montagna report grant SCA145102 from the Strategy for Patient Oriented Research (SPOR) during the conduct of the study. L.S. Stone reports grants and nonfinancial support from Quebec Pain Research Network during the conduct of the study. The remaining authors have no conflicts of interest to declare.

Previous presentations: Quebec Pain Research Network Annual General Meeting November 2017—Project update, Quebec Pain Research Network Annual General Meeting January 2019—Project update, Canadian Pain Network Biomarkers Meeting—January 2019—Project overview.

## Appendix A. Supplemental digital content

Supplemental digital content associated with this article can be found online at http://links.lww.com/PR9/A59.
